# Meta-linguistic awareness skills in Chinese-speaking children with hyperlexia: A single-case study

**DOI:** 10.3389/fpsyg.2023.1049775

**Published:** 2023-02-22

**Authors:** Lirong Luo, I-Fan Su

**Affiliations:** Human Communication, Development and Information Sciences, The University of Hong Kong, Pokfulam, Hong Kong SAR, China

**Keywords:** hyperlexia, reading, meta-linguistic awareness, reading in Chinese, ASD

## Abstract

**Purpose:**

The aim of the study is to examine the meta-linguistic awareness skills contributing to reading aloud in a Chinese-speaking child with hyperlexia.

**Methods:**

Case study approach was used with one case of hyperlexia (TYH) and two control groups: typically developing (TD) children matched for chronological age (CA) and TD children matched for mental ability (MA). A battery of phonological, morphological, and orthographic awareness skill tests were administered.

**Results:**

Results from the modified *t*-test found that the hyperlexic child did not demonstrate advanced meta-linguistic awareness skills in comparison with the two control groups. On the contrary, TYH’s morphological awareness skills were even lower than the CA control group. Also, in the orthographic awareness test, TYH demonstrated weaker knowledge of character structure and components than the two control groups although his ability in the recognition of real words is intact. In addition, the predictability of orthographic awareness skill was comparable to the CA group with predicted score showed no difference to his obtained score, while TYH achieved a significantly higher reading score than what his morphological awareness skills should predict with reference to TD children of similar age; as well as what his phonological awareness skill predict with reference to the MA group.

**Conclusion:**

The findings suggest that TYH can achieve advanced reading ability with comparable phonological and orthographic awareness skill, despite his weakness in morphological awareness. It is concluded that the hyperlexic reading in Chinese might be achieved through the direct mapping between the whole character and the sound.

## 1. Introduction

Children with hyperlexia have superior word recognition ability despite impaired comprehension (Nation, 1999), but it remains unclear if the meta-linguistic awareness skills support reading in hyperlexia. Previous studies have found that reading aloud is supported by several meta-linguistic awareness skills, including phonological awareness skills (knowledge of the phonological structure and the ability to manipulate speech sounds), morphological awareness skills (knowledge and the ability to make use of morphemes), and orthographical awareness skills (knowledge of the structure of written words), which are important to successful reading and comprehension (Nagy and Anderson, 1999; McBride-Chang and Ho, 2000; McBride-Chang et al., 2004; Tong et al., 2009; Li et al., 2012; Tong et al., 2017). However, it is unclear how meta-linguistic awareness skills contribute to reading in Chinese-speaking hyperlexics. In order to further understand the advanced reading ability in hyperlexic children and the linguistic skills that support their superior reading ability, the current study aims to examine the reading ability of children with hyperlexia in relation to their phonological, morphological and orthographical awareness skills.

Previous studies in alphabetic languages have investigated how hyperlexic children read by looking at the reading of real words, pseudowords, and nonwords. It was found that children with hyperlexia achieve higher scores in reading real words (Healy, 1982; Castles et al., 2010) compared with their age-matched peers or compared to their own comprehension ability. Apart from real words, children with hyperlexia are also able to read out pseudowords and nonwords that do not exist, which has been argued to show that they can make use of the Grapheme Phoneme Correspondence (GPC) rules during reading aloud. This has led some to suggest that children with hyperlexia might be skilled in phonological awareness and processing (Kennedy, 2003).

However, studies have also found that children with hyperlexia do not rely solely on the GPC rule while reading, and lexical representations also play an important role. For instance, Glosser et al. (1996) investigated the reading of pseudowords that are analogous to real words (e.g., pelt, drowl, and nove) and pseudowords that have no real word analogy (e.g., fwov) in a child with hyperlexia and 10 reading ability-matched children. They found comparable performance in reading of pseudowords that have real word analogy, whereas the hyperlexic child achieved a lower score for pseudowords with no real word analogy when compared with the reading ability-matched control. The result suggests that word reading in hyperlexic children speaking English may rely more on the retrieval of lexical representations compared with typical developing children matched for reading ability, and that phonological access is achieved using lexical representation instead of the GPC mapping. This may imply that hyperlexic reading does not solely rely on the mapping between letters and sounds (Castles et al., 2010). In sum, apart from using GPC rules in reading, lexical knowledge which facilitates reading through analogy is likely to play an important role in hyperlexic reading, suggesting lexical processing of phonological and orthographic representations are possible strengths in hyperlexic reading.

Previous studies from typically developing children found close relationship between reading ability and meta-linguistic awareness skills in both alphabetic languages like English and morpho-syllabic language such as Chinese (e.g., Siegel and Ryan, 1988; Tong and McBride-Chang, 2010a,b; Li et al., 2012; Liu et al., 2017). Studies in alphabetic languages found a significant positive correlation between word recognition and phonological skills, and children with reading disability were found to have a core deficit in phonological processing (Siegel and Ryan, 1988), suggesting that phonological awareness is important to word decoding and reading in alphabetic languages. In Chinese, apart from phonological awareness skill, morphological and orthographic awareness skills were found to also play a significant role in reading (McBride-Chang et al., 2004, 2008; Tong et al., 2009; Tong and McBride-Chang, 2010a,b; Li et al., 2012).

Although morphological and orthographic awareness skills were not found to be a strong predictor of reading ability in alphabetic languages, we still included them in this study considering the features of the non-alphabetic language like Chinese. Namely, written Chinese is represented by characters and each character is pronounced as a one-syllable sound, the mapping between character and its pronunciation is not as regular as in alphabetic languages which means that it is difficult to infer the pronunciation and lexical tone of a character by its orthography alone (Law et al., 2005). And that each Chinese character carries meanings and can be compounded to form new words, which means that characters are not only the basic unit of sound, but also morphemes. These features of the Chinese script warrant a need to further explore the role of morphological and orthographic awareness skill in Chinese hyperlexic reading as possible meta-linguistic awareness differences could be found to support hyperlexic reading.

Returning to hyperlexia, few studies have looked at meta-linguistic awareness skills in these children and mixed findings were reported, with most reported cases on alphabetic language speakers. Newman et al. (2007) examined phonological awareness skills in English-speaking children with ASD and hyperlexia using tasks in which children were asked to rhyme, detect sounds in words, and produce new words by substituting a sound in words or reversing the sound in words. Interestingly, Newman found no significant differences between ASD children with hyperlexia (ASD + HPL), typical developing children matched for single word reading level (TD), and ASD children without hyperlexia (ASD-HPL), suggesting that hyperlexic children do not have superior phonological awareness skills than typical developing or other ASD children. Different results were found in Cardoso-Martins and Da Silva (2010), which also looked at phonological awareness skills in Portuguese-speaking hyperlexics. In the study, children were asked to identify the word that starts with the target consonant among three choices, children with hyperlexia and ASD (ASD + HPL) performed worse in this phonemic awareness task compared with ASD children without hyperlexia (ASD-HPL) and typically developing children matched for reading ability (TD). Note however that in the two studies, the ASD + HPL group were identified based on parent reports of “exceptional and precocious reading of single words,” instead of formal standardized assessment of reading and language related skills, which may result in the different findings of the two studies. Sparks (1995) also found phonemic awareness skills in 3 hyperlexic children to be lower than what would be expected based on their reading ability. A follow-up study of the two hyperlexic children 7 to 8 years later (Sparks, 2001) found that although their phonemic awareness skills improved over time, they were still below their grade level. These findings are consistent with Cardoso-Martins and Da Silva (2010), but are challenging to be generalized to Chinese hyperlexic reading as the study only focused on specific phonological awareness at the phonemic level, and different tasks were used for the assessment. However, phonological awareness is comprised more than phonemic awareness (e.g., phonological processing at the syllable level) and given that Chinese characters are syllable-based (one syllable for one character), it remains an open question whether Chinese hyperlexics will show comparable or weaker phonological awareness skills at the syllable and phonemic level. Overall, these results reflect that the phonological awareness skills in hyperlexic children are not higher than their age-matched peers or reading ability-matched typically developing peers, which may suggest that their advanced word reading skill may not depend on having superior phonological awareness skills. However, the studies only compared hyperlexic children with TD children of similar reading ability, which might be biased considering that hyperlexic children are often on the autism spectrum disorder (ASD), meaning that the TD children matched for reading ability might have a higher mental ability advantage. Although the hyperlexic children were not found to show advanced phonological skill compared to TD children who can read at a similar level, further comparison with TD children matched for chronological age or mental ability is needed.

In terms of orthographic awareness skill, Sparks (2001, 2004) looked at the orthographic awareness skills of English-speaking children with hyperlexia using a word decision task, in which children were presented with 17 pairs of pseudowords, whereby one of them was orthographically regular and the other irregular (i.e., containing the letter combinations that never appear in English words). The children were asked to decide which stimuli could be a word. The result found that the six cases of hyperlexia exhibited orthographic awareness skill within average range or lower than their reading ability. However, the study did not include a control group (Sparks, 2001) or only had one typical developing control child for each case (Sparks, 2004). This makes it difficult to make comparison between hyperlexic children and typical developing peers. More studies are needed to provide evidence on orthographic awareness skills in hyperlexic children to understand the core meta-linguistic awareness skills underlying superior reading. Also, there is currently no study looking at orthographic awareness (or morphological awareness) skills in Chinese-speaking hyperlexics, and therefore it is not clear whether the results from alphabetic languages can be generalized to Chinese hyperlexics as orthographic awareness skills are not considered core skills in English but remain important for typically developing Chinese young readers.

Although previous study has not looked at morphological awareness skills in hyperlexic children, weakness in language comprehension observed in children with hyperlexia (Nation, et al., 2006; Newman et al., 2007) could shed light on how children with hyperlexia would perform in morphological awareness tasks. Given that morphological awareness skills would require knowledge of meaning or semantic information of the lexical or sub-lexical representations and the ability to manipulate meanings, it is likely that children with hyperlexia will show weakness in morphological awareness skills. In terms of hyperlexic children speaking a non-alphabetic language like Chinese where there is no reliable GPC rule to capitalize on and whose semantic system is likely to be weak, it postulated that children with hyperlexia speaking Chinese may rely on the direct retrieval of lexical representation of words as found in English-speaking hyperlexics (Glosser et al., 1996).

Adopting a case study approach, this study examined the meta-linguistic awareness skills of a hyperlexic child (TYH) with ASD including phonological, morphological, and orthographic awareness skills. Despite that children with hyperlexia are good at reading, the profile of their meta-linguistic awareness skills has not been systematically investigated, and it remains unclear whether Chinese-speaking children with hyperlexia also have good meta-linguistic awareness skills. Furthermore, predictors of reading in Chinese differ from English with greater influence from morphological awareness skills; therefore, it is also unknown whether the underlying meta-linguistic awareness skills that contribute to advanced reading ability in hyperlexic children also differ or not. Previous findings suggest that hyperlexic children can achieve superior reading despite weakness in orthographic skills, which may be because orthographic awareness skills do not play an important role in the reading of alphabetic languages. However, in the case of reading Chinese, where orthographic awareness skills are of similar or more importance to reading as phonological awareness skill, it is not clear how advanced reading is supported by orthographic awareness skill.

Therefore, the current study attempts to answer two research questions: (1) How does the Chinese-speaking hyperlexic child perform in phonological, morphological and orthographic awareness skills tests compared with TD children of similar age (CA), and with similar mental ability (MA)? And (2) does meta-linguistic awareness skills predict reading ability in the hyperlexic child to the same degree as TD children of similar age (CA), and TD children with similar mental ability (MA)?

It is predicted that the general performance on phonological awareness skill of the hyperlexic child is comparable to the CA group who are of similar age to the hyperlexic child, and better than the MA group (Newman et al., 2007; Cardoso-Martins and Da Silva, 2010). For the morphological awareness skills, the performance of the hyperlexic child is predicted to be lower than the CA group, and comparable to the MA group given that children with hyperlexia may have difficulties manipulating morphemes due to their difficulty in language comprehension and extracting meaning from words. For orthographic awareness skill, it is hypothesized that the hyperlexic child would perform better or comparable than the CA and the MA group, given that orthographic awareness skill is an important predictor of reading ability in Chinese (Tong et al., 2009; Tong and McBride-Chang, 2010b). For the second research question, it is expected that the meta-linguistic awareness skills predict reading of TYH similarly as the CA group who are of similar age, but differently compared with the MA group who are around 3 years younger than the hyperlexic child. Namely, when compared to children of similar mental ability, TYH’s actual reading ability might be higher than the predicted reading ability estimated by his meta-linguistic awareness skills when compared to children of similar mental ability.

## 2. Methods

### 2.1. Subjects

The current study adopted case study design, including one hyperlexia case and two control groups matched for chronological age (CA group) and mental ability (MA group).

#### 2.1.1. The hyperlexic child

The hyperlexia case TYH (9;05) was a 9-year-old native Cantonese-speaking boy studying in a main-stream school grade 3 in Hong Kong. He was diagnosed with Autism Spectrum Disorder (ASD) at the age of 2 years 3 months. His parents reported that TYH started reading at the age of 5 without formal instruction, and had a special interest in written words and symbols, and often liked to sound out words in the environment. According to the standardized test results, TYH demonstrated more than 3 years of discrepancy between single character reading ability (2 SD higher than his grade level) and oral language comprehension ability (around 3 years behind) according to the normative data (see Table 1). The reading pattern observed in TYH also fit into the 4-feature definition proposed in Ostrolenk et al. (2017) and the definition proposed in Mottron et al. (2013).

**Table 1:** Non-verbal IQ and standardized language assessment results of TYH and comparison with his chronological age level.

	Test	Max score	TYH			
			Raw score	Standard score	Percentile	Age equivalent
Age (year)			9;05			
Non-verbal IQ	CPM	36	22	80	9%	
Single character reading	HKGCNT grade 3	150	140	2 (*z*-score)	95.3%	
Sentence comprehension	HKCOLAS grammar comprehension subset	65	31	<−3	<0.1%	5;04–5;07
Paragraph comprehension	HKCOLAS paragraph comprehension subset	N/A	10	−3	0.1%	5;08–6;07

CPM, Raven’s Colored Progressive Matrices; HKGCNT, Hong Kong Graded Character Naming Test; HKCOLAS, Hong Kong Cantonese Oral Language Scale.

#### 2.1.2. Control groups

The chronological age-matched group (CA group) consisted of 12 typically developing children (*M*_age_ = 9.01, SD = 0.76, male: 10, female: 2) studying in main-stream schools in Hong Kong. The mental ability-matched group (MA group) included 14 typical developing children (*M*_age_ = 6.04, SD = 0.28, male: 6, female: 8) matched with TYH in non-verbal IQ score, and were about 3 years younger than TYH.

### 2.2. Material

#### 2.2.1. Non-verbal IQ assessment

Raven’s Colored Progressive Matrices (Raven and Court, 1998) was administered to assess children’s non-verbal intelligence.

#### 2.2.2. Language assessments

##### 2.2.2.1. Character reading test

The Grade 1 and Grade 3 Hong Kong Graded Character Naming Test (HKGCNT) (Leung et al., 2008) was administered to the children participated in the study.

##### 2.2.2.2. Word reading task

Children were asked to read out 65 words from the Hong Kong Cantonese Receptive Vocabulary Test (HKCRVT) (Lee et al., 1996).

##### 2.2.2.3. CRVT spoken word-picture matching test

Sixty-five spoken word-picture matching test items (Lee et al., 1996) were administered to assess children’s word listening comprehension ability.

##### 2.2.2.4. CRVT written word-picture matching test

Children were asked to select from 4 choices of pictures the one that match the target written word shown on the center of the screen. 65 test items from the HKCRVT (Lee et al., 1996) were administered.

##### 2.2.2.5. Sentence and paragraph comprehension test

Children’s comprehension ability in sentences and paragraphs was assessed using the Grammar Comprehension and the Paragraph Comprehension parts of the Hong Kong Cantonese Oral Language Scale (HKCOLAS) (T’sou et al., 2006). As different age-appropriate materials were used for children of different age range in the paragraph comprehension part, standardized scores were used for comparison.

#### 2.2.3. Meta-linguistic awareness tests

##### 2.2.3.1. Phonological awareness tests

The phonological awareness tests aimed at assessing the knowledge of the sound structures of Cantonese using an onset deletion and syllable deletion task, including 29 syllable deletion test items and 22 onset deletion test items from Tong et al. (2017) and Tong and McBride-Chang (2010b). In the syllable deletion test, the experimenter orally presented a three-syllable word and participants were asked to say the word out after taking out the first, second, or the third syllable (e.g., Please say electrical bike, now please say electrical bike without saying electrical. - bike). In the onset deletion task, the child was asked to say a word they heard without the initial consonant (e.g., Please say/so1/without saying the first sound. - /o1/).

##### 2.2.3.2. Morphological awareness tests

Originally developed by Tong et al. (2017) and Tong and McBride-Chang (2010b), three tasks were included in the current study: homographic discrimination, morphological construction, and morpho-grammar derivation. In the homographic discrimination task, children were presented orally with 4 bi-syllabic words that contain the same character and were asked to pick out the words that contain the homographic character of different meaning from the other three. There are 34 items in this task, and the accuracy of the response was recorded.

In the morphological construction task, the experimenter orally created a three or four-sentence scenario and asked the child to create Chinese words according to the scenario (e.g., This paper is white, we call it white paper, and if I have a paper in red color, how would you call it?). This task had 27 items, and the accuracy of the oral response was recorded.

In the morpho-grammar deviation task, the experimenter orally presented a sentence with a word missing from it and a target word related to the missing word, and children were asked to come up with a word to fill in the sentence and make the sentence grammatical and complete. If the child could not produce a word by themselves, 4 words were given to choose from. Accurate responses are words that are related to the target word and also in line with the meaning of the sentence. Same marks were given for correct choice and self-generated response. There were 28 items in total, and the accuracy of oral responses was recorded.

##### 2.2.3.3. Orthographical awareness test

In this test, a Chinese character judgment task was administered to the participants. The stimuli include 60 real Chinese characters as fillers, 19 pseudo characters, and 60 non-characters. As shown in Table 2, pseudo characters were constructed using a combination of real radicals in their legal position to look like real characters. Non-characters were either real radicals in the wrong position, non-existing radicals in the correct position (the position of the original radical), or non-existing radicals in the wrong position. Non-existing radicals were created through insertion or deletion of strokes or mirroring the logographeme or radical. Real character stimuli are matched for radical frequency, stroke number with the pseudo and non-characters stimuli. Words and nonwords were visually presented to the child, and they were given two paper cards with a tick or a cross on it. Children were instructed to hold up the tick card if they thought the character was a real character, and to hold up the paper card with a cross if the child thought it was a fake character. Paper cards were used to so that no oral response was needed. Five practice trails were given before the test. This task was presented using the DMDX program (Forster and Forster, 2003), and the response accuracy were recorded.

**Table 2:** Features and examples of stimuli in the orthographic awareness test.

Stimuli	No.	Real character	Existing radical	legality of radical position	Existing logographeme	Example	
Real Chinese characters	60	Yes	Yes	Yes	Yes	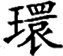	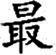
Pseudo characters	19	No	Yes	Yes	Yes	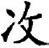	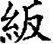
Non-characters	20	No	Yes	No	Yes	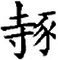	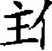
	20	No	No	Yes	No	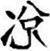	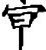
	20	No	No	Yes	Yes	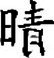	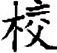

Stimuli in real radical are matched for radical frequency, stroke number with the pseudo and non-characters condition.

### 2.3. Procedure

Participants were recruited by sending invitation mails and emails to schools, parent groups, and community centers. Children took the tests either in their school, the community center, or in the Lab rooms in the university. Children completed all assessments and meta-linguistic awareness tests within the three 90-min sessions. The order of the tasks was arranged to maintain the child’s motivation level.

### 2.4. Data analysis

Raw scores of the phonological and morphological awareness tests were used for the comparison between the hyperlexic child and the control groups. The d-prime score was used for the overall performance in the orthographic awareness skill. The d-prime score provides a measurement on how participants correctly accept the real and pseudo characters that follows the orthographic rule, and reject the non-characters that contain an ill-formed radical or components or/and in an illegal position, without the interference of response bias. The d-prime score was calculated by the difference between the Hit rate (respond “yes” to real word and pseudoword stimuli) and the False Alarm rate (respond “yes” to nonword stimuli) was done by applying the “NORMSINV” function in excel. The false alarm of 0 has been replaced with 0.5 divided by 60 (total number of nonword stimuli) for the calculation as suggested in Stanislaw and Todorov (1999) and Macmillan and Kaplan (1985).

Three steps of statistical analyses were developed and implemented using the computer program “Singlims_ES” (Crawford et al., 2010) and “REGBUILD” (Crawford and Garthwaite, 2007). In order to determine whether children with hyperlexia showed better performance in phonological, morphological or orthographic awareness skills, in the first step, the scores of the three meta-linguistic awareness tasks were compared between TYH and the three control groups using the modified *t*-test (Crawford and Howell, 1998; Bi et al., 2009; Han et al., 2011) incorporated in the computer program “Singlims_ES” (Crawford et al., 2010). In the second step, hierarchical regression models were constructed for each control group (CA and MA) to establish which meta-linguistic awareness skills predicted reading aloud ability. In the last step, using the meta-linguistic awareness skills that significantly predicted reading ability in the control groups derived from step two, a regression equation was built on the predictor and the reading score. The reading score of TYH was subsequently compared with the predicted score based on the regression equation to investigate whether that particular meta-linguistic awareness skill predicts reading ability to a similar degree in hyperlexic children when compared with CA and MA control groups (Han et al., 2013). The last step was performed by using “REGBUILD” program (Crawford and Garthwaite, 2007).

## 3. Results

Word reading and character reading scores of TYH and control groups are presented in Table 3. The CA group had a significantly higher non-verbal IQ than TYH (*p* = 0.02, see Table 3), No difference was found in the performance of TYH and the CA group, and TYH outperformed the MA group with marginal significance in the word reading test.

**Table 3:** Age, non-verbal IQ, and reading ability measures of control participants with comparison to TYH.

Measurement (maximum score)	TYH	CA (*N* = 12)			MA (*N* = 14)		
		*M*	*SD*	Sig(*p*)	*M*	*SD*	Sig(*p*)
Age	09;05	09;01	0.76	0.338	06;04	0.28	<0.001***
Non-verbal IQ (36)	22	30.92	3.70	0.020*	26.79	5.35	0.202
Word Reading (CRVT) (65)	64	60.67	5.03	0.269	33.79	19.52	0.079+
Single character reading (GCNT_P3) (150)	140	115.83	22.79	0.165			
Single character reading (GCNT_P1) (150)					58.86	38.45	

CRVT, Hong Kong Cantonese Receptive Vocabulary Test; HKGCNT, Hong Kong Graded Character Naming Test; +: *p* < 0.1, *: *p* < 0.05, **: *p* < 0.01, ***: *p* < 0.001, one-tailed.

### 3.1. Comparison of meta-linguistic awareness skills in TYH and control groups

Scores of phonological, morphological, and orthographic awareness tests were compared between TYH and the control groups using modified *t*-test (*t_modified_*) (see Table 4), and among the control groups using the parametric independent sample *t*-test or non-parametric Mann–Whitney U test when the assumptions of normality were violated.

**Table 4:** Phonological, morphological, and orthographic awareness skills of participants and comparison with TYH.

Measurement (maximum score)	TYH	CA (*N* = 12)			MA (*N* = 14)		
		*M*	*SD*	Sig(*p*)	*M*	*SD*	Sig(*p*)
Phonological awareness composite score (51)	36	35.17	9.39	0.467	31.79	8.28	0.315
Syllable deletion (29)	27	27.83	1.53	0.306	26	3.06	0.378
Onset deletion (22)	9	6.58	8.88	0.399	5.79	6.61	0.323
Morphological awareness composite score (89)	47	63.17	8.89	0.054+	40	10.6	0.267
Homophone discrimination (34)	9	19.50	3.61	<0.009**	11.21	3.91	0.297
Morphological construction (27)	20	22.75	2.22	0.130	15.93	4.46	0.197
Morpho-grammar deviation (28)	18	20.92	4.56	0.275	12.86	3.84	0.109
Orthographic awareness D-prime score	1.43	2.46	0.81	0.119	1.52	0.46	0.404
Real words (60)	57	54.50	3.53	0.255	47.36	6.85	0.098+
Pseudowords and nonwords (79)	49	62.92	4.76	0.008**	58.21	5.38	0.061+
Pseudowords (19)	8	8.67	5.03	0.450	10.71	2.87	0.189
Nonwords (60)	41	54.25	4.05	0.005**	47.5	5.37	0.132

+: *p* < 0.1, *: *p* < 0.05, **: *p* < 0.01, ***: *p* < 0.001, one-tailed.

For the phonological awareness skill, no significant difference was found in the composite score between the CA and MA control groups (*U* = 65, *p* = 0.326, *Z* = −0.98). There is also no difference found in the syllable deletion task (*U* = 54, *p* = 0.110, *Z* = −1.60) and the onset deletion task (*U* = 81, *p* = 0.871, *Z* = 0.16). The overall performance of TYH did not differ from the two control groups in phonological awareness test (CA: *t_modified_* = 0.09; MA: *t_modified_* = 0.49). For the syllable and onset deletion tasks (see Table 4), comparable performances were found on the two subtests between the two control groups and TYH (Syllable deletion: CA: *t_modified_* = −0.52; MA: *t_modified_* = 0.32; Onset deletion: CA: *t_modified_* = 0.26; MA: *t_modified_* = 0.47). The results suggest that TYH’s phonological awareness skill is comparable to children matched for age, and TYH outperformed children of similar mental ability, but the difference did not reach significance level.

As for the morphological awareness skill, the CA group achieved significantly higher score than the MA group in the overall composite score (*U* = 8.50, *p* < 0.001, *Z* = −3.89) as well as in the three subtests: Homophone Discrimination (*U* = 8.50, *p* < 0.001, *Z* = −3.84), Morphological Construction task (*U* = 13, *p* < 0.001, *Z =* −3.68), and Morpho-grammar Deviation tasks (*t* (24) = 4.89, *p* < 0.001). Generally, TYH’s composite morphological awareness score was marginally lower than the CA group (*t_modified_* = −1.75), but comparable to the MA group (*t_modified_* = 0.64), suggesting that his morphological awareness skill may not be as strong as his reading ability. Looking further into the different morphological tasks (see Table 4), TYH scored significantly lower in Homophone Discrimination than the CA group (*t_modified_* = −2.80), suggesting weaker skills in differentiating the different meanings of a character in word context, and no difference was found in Morphological Construction (*t_modified_* = −1.19) and Morpho-grammar Deviation tasks (*t_modified_* = −0.62) in comparison with the CA group. No difference was found in the comparison with the MA group in the subtests (*t_modified_* = −0.55; *t_modified_* = 0.88; *t_modified_* = 1.29 respectively).

Overall performance on the orthographic awareness task was evaluated using d-prime score which indicates how well children can discriminate well-formed characters from non-characters. The CA group showed higher accuracy in accepting real and pseudo words (as they both conform to the orthographic rules) and rejecting nonwords that violated orthographic rules than the MA group with higher d-prime score (*t* (24) = 6.68, *p* = 0.001), suggesting that with age, typically developing children gain better knowledge on the structure of Chinese characters in Grade 3 compared with children in Grade 1. The overall performance of TYH (refer to Table 4) was comparable to the CA (*t_modified_* = −1.25) and MA groups (*t_modified_* = −0.25), indicating that generally TYH also did not show any advantage in the general orthographic knowledge compared with CA and MA control groups.

As for the non-existing stimuli (pseudowords and nonwords) that assess children’s knowledge of character structure and components without interference of existing characters, CA group outperformed the MA group in non-existing stimuli (*t* (24) = 2.34, *p* = 0.028), and TYH performed worse than the CA group (*t_modified_* = −2.81), and marginally lower than the MA group (*t_modified_* = −1.65), indicating lower performance in accepting well-formed components in legal position and rejecting ill-formed components in illegal position. The CA group also are less likely to have false alarms and accept nonword stimuli as “looks like a real word” than the younger children with the CA group outperforming the MA group in the nonword stimuli (*t* (24) = −1.25, *p* = 0.023). More false alarms were observed in TYH in the nonword stimuli compared with the CA group (*t_modified_* = −3.145) while no difference was found compared with the MA group (*t_modified_* = −1.168). These results suggest TYH may have difficulty rejecting nonword stimuli with ill-formed components or radical in illegal position compared with children of similar age. For the pseudowords, no difference was found between the two control groups (*U* = 59.5, *p* = 0.203, *Z* = −1.27), and comparable performance was observed in TYH compared with the two control groups (CA group: *t_modified_* = −0.13; MA group: *t_modified_* = −0.92), indicating that TYH does not have difficulty accepting the pseudoword stimuli that has radicals that overlap with exiting real characters, while weaker in rejecting stimuli that contain ill-formed components or radical in illegal position. For the identification of real word stimuli which may require knowledge of the existence of real characters, the CA group outperformed the MA group (*t* (24) = 3.41, *p* = 0.003), and TYH scored marginally higher than the MA group (*t_modified_* = 1.36), while no difference was observed in the comparison with the CA group (*t_modified_* = 0.68), suggesting TYH has better whole word recognition compared with children with similar mental ability while this advantage was not salient when compared with children of similar age.

Taken together, the overall orthographic awareness skill of TYH appears within the normal range of the typically developing CA and MA groups. However, his knowledge of the orthographic rules of Chinese characters was found to be weaker than the CA group and comparable to the MA group which mainly driven by the difficulty in rejecting illegal stimuli. TYH also has better lexical recognition of whole characters than children of similar mental ability.

In summary, with reading aloud ability similar to the CA group and better than the MA group, TYH showed no difference in phonological awareness skill compared with the control groups, while his morphological awareness skill was weaker than the CA group and comparable to children with similar mental ability (MA group). In addition, no difference was found in his orthographic awareness skill compared to the CA and MA groups, but after taking his exceptional lexical recognition skills into consideration, knowledge of the orthographic structure of Chinese characters was found to be lower than the CA group. These findings indicate that although TYH is good at reading aloud, his meta-linguistic awareness skills are not as advanced as his reading ability.

### 3.2. Predictors of reading ability in the control groups

Two-step hierarchical regression models were constructed for the control groups to understand whether these meta-linguistic awareness skills can predict word and character reading. The dependent variable was the reading composite score, which was calculated by the summation of the grade-corrected z-score of the GCNT single character reading test (the z-score of individual children in relation to their grade level based on the normative data), and the z-score of the CRVT word reading in the corresponding control group (CRVT z-score of children in the MA/CA group was calculated by the subtraction of individual score and the mean score of the MA/CA group divided by the standard deviation; the CRVT z-score of TYH was calculated by the subtraction of individual score and the mean score of the CA group divided by the standard deviation). The reading composite score was used as an indicator of general reading ability which includes both single character reading and word reading in Chinese. In the first step, chronological age (in month) and the non-verbal IQ score were entered into the model as control factors (Table 5). In the second step, the Phonological Awareness composite score, Morphological Awareness composite score, and the d-prime score of the Orthographic Awareness test were entered using the stepwise method.

**Table 5:** Hierarchical regression explaining reading ability (reading composite score) from age, non-verbal IQ, and morphological awareness skills for the CA group.

CA group										MA group									
Step	Predictor	Unstandardized coefficient		Standardized coefficient		*R^2^*	*R*^2^ change	*F*	*p*	Step	Predictor	Unstandardized coefficient		Standardized coefficient		*R^2^*	*R^2^* change	*F*	*p*
		*B*	*SE*	*β*	*p*							*B*	*SE*	*β*	*p*				
1						0.51	0.51	4.68	0.040*	1						0.32	0.32	2.54	0.124
	Age	0.16	0.06	0.69	0.017						Age	0.24	0.20	0.34	0.241				
	CPM	0.06	0.14	0.11	0.657						CPM	0.15	0.12	0.34	0.242				
2						0.83	0.32	14.94	0.005**	2						0.71	0.40	13.64	0.004**
	Age	0.12	0.04	0.51	0.011						Age	0.08	0.14	0.10	0.608				
	CPM	−0.10	0.10	−0.17	0.346						CPM	0.08	0.09	0.17	0.387				
	MA	0.16	0.04	0.67	0.005						PA	0.21	0.06	0.71	0.004				
3						0.92	0.09	7.49	0.029*										
	Age	0.15	0.03	0.66	0.001														
	CPM	−0.15	0.07	−0.26	0.082														
	MA	0.14	0.03	0.57	0.004														
	OA	0.93	0.34	0.35	0.029														

CPM, Raven’s Colored Progressive Matrices; MA, morphological awareness; PA, phonological awareness; OA, orthographic awareness. +: *p* < 0.1, *: *p* < 0.05, **: *p* < 0.01, ***: *p* < 0.001, one-tailed.

As shown in Table 5, after controlled for chronological age and non-verbal IQ, morphological awareness skills and orthographic awareness skills significantly predict general reading ability in the CA group (*p* = 0.005 and *p* = 0.029 respectively; *R^2^* = 0.92). In the MA group, only phonological awareness skill was a significant predictor of reading ability (*p* = 0.004), and significantly improved the fit of the model with *R^2^* = 0.71 (see Table 5).

### 3.3. Meta-linguistic awareness skills in the reading of TYH

Based on the findings from the CA and MA groups, linear regression equations were built to predict reading ability by morphological awareness skill, orthographic awareness (for the CA group) and phonological awareness skill (for the MA group). The obtained reading composite score of TYH was then compared with his predicted score calculated by using the hierarchical regression equation based on the summary data from the control groups (Crawford and Garthwaite, 2007).

#### 3.3.1. Morphological awareness

The role of morphological awareness in the reading performance in the hyperlexic child was examined in comparison with the CA group. As shown in Table 6, the obtained reading composite scores of TYH were found to be significantly higher than the predicted score from his morphological awareness skill based on the regression equation (*p* = 0.005). The result indicates that TYH has a higher reading ability than what his morphological awareness skill can predict, meaning that the predictability of morphological awareness is significantly different from the CA group. In another word, given that better morphological awareness skills are needed in the CA group to achieve the same reading ability as TYH, morphological awareness skills in hyperlexic reading may not be as important to support reading as in age-matched TD children.

**Table 6:** The comparison of obtained reading composite score in TYH and the predicted score by meta-linguistic awareness skills based on the equation built on data from the CA and MA groups.

Independent variable	Dependent variable	Group	Regression equation		TYH		Comparison with predicted score		
			Slope	Intercept	Obtained score	Predicted score	Standard error of estimate	Standardized discrepancy	Sig(*p*)
MA raw score	Reading composite score	CA	0.18	−11.52	2.66	−2.88	1.47	3.21	0.005**
OA d-prime score		CA	0.58	−1.35	2.66	−0.53	2.20	1.30	0.111
PA skill raw score		MA	0.14	−5.64	2.66	−0.43	0.87	3.40	0.003**

CPM, Raven’s Colored Progressive Matrices; MA, morphological awareness; PA, phonological awareness; OA, orthographic awareness. +: *p* < 0.1, *: *p* < 0.05, **: *p* < 0.01, ***: *p* < 0.001, one-tailed.

#### 3.3.2. Orthographic awareness

According to the results (Table 6), the reading ability predicted by orthographic awareness skills in TYH showed no difference from his obtained score based on the regression equation build based on the performance of the CA group (*p* = 0.111), indicating the orthographic awareness skill can predict reading in the hyperlexic child to the similar degree as in TD children matched for chronological age.

#### 3.3.3. Phonological awareness

When referring to the equation built based on data from the MA group (Table 6), the obtained score of TYH was significantly higher than the predicted score by phonological awareness skills (*p* = 0.003). This suggests that phonological awareness skill predicts word reading in TYH differently from TD children matched for mental ability, and TYH achieved higher reading ability than what his phonological awareness skill can predict with reference to TD children matched for mental ability. This indicate that to achieve TYH’s word reading ability, a lower phonological awareness performance is needed in TYH than the MA group.

## 4. Discussion

The current study examined the meta-linguistic awareness skills in a Chinese-speaking child with hyperlexia. The study found that in general, the performance of the hyperlexic child was comparable to the CA and MA control groups, except that his morphological awareness skill was found to be lower than the CA matched TD children. The findings suggest that superior reading aloud ability may not necessarily support by advanced knowledge of phonological, morphological and orthographic structures of Chinese. In addition, the hyperlexic child’s orthographic awareness skill was of similar importance to reading ability as in TD children of similar age, while morphological awareness and phonological awareness skill predict reading differently in TYH in comparison with the CA and MA group, respectively. The findings suggest that TYH can read significantly better than what his morphological awareness can predict compared with CA matched TD children, and higher than the score predicted by his phonological awareness skill compared with TD children matched for mental ability.

Although there are previous studies reported cases of hyperlexia who exhibited strength in the grapheme to phoneme mapping, phonological processing and orthographic processing (e.g., Kennedy, 2003), there has not been much direct evidence of advantage in meta-linguistic awareness reported in hyperlexic children in alphabetic languages.

Looking at phonological, morphological and orthographic awareness skills separately, similar to previous findings in alphabetic languages that phonological awareness skill at phoneme level is well developed in children with hyperlexia (Newman et al., 2007; Cardoso-Martins and Da Silva, 2010), the Chinese-speaking case showed comparable skills in both phoneme and syllable level to TD children matched for chronological age and children of similar mental ability, demonstrating well-developed skill in manipulating basic sound unit in Chinese.

It has been found that morphological awareness skills play an important role in both reading aloud and language comprehension in Chinese. However, morphological awareness might be challenging for children with hyperlexia especially in task that require differentiating meanings of homophones based on the word context. This finding echoes Wong et al. (2013) and Wong (2013) which found Chinese hyperlexic children have difficulties reading homographic heterophones (characters that have different pronunciation depending on context) in word context despite their advanced reading ability and this difficulty might come from the weakness in discriminating different meanings of homophones. Together these findings add to previous knowledge that Chinese-speaking children with hyperlexia may have difficulties in making use of context to discriminate different meanings of the same morpheme, and this may also relate to the difficulty in language comprehension skills (Deacon et al., 2015). Few study has looked at morphological awareness skills in children with hyperlexia in alphabetic language, it is not clear whether this is a feature specific to Chinese or not. Whether weaknesses in morphological awareness also present in hyperlexia speaking alphabetic languages yields further investigation.

Findings from the orthographic awareness tests seem to imply that TYH may simply rely on his lexical knowledge when making decisions on whether stimuli looked like a real character or not, instead of extracting and making use his knowledge on orthographic structure. His poorer performance on non-existing stimuli also supports the notion that the hyperlexic child may process the Chinese characters as a whole, and does not attend to the components of characters as much as the TD children when making the judgment. In other words, children with hyperlexia differ from typically developing children in the (lack of) use of sub-lexical orthographic knowledge. Although the hyperlexic child demonstrated weakness in the knowledge of orthographic rules, he exhibited strengths in recognizing real characters stored in his orthographic lexicon compared with TD children matched for mental ability. This may indicate that the hyperlexic child process the characters as a whole instead of by components. This finding suggests that reliance on lexical knowledge (Glosser et al., 1996) might be a language universal feature of hyperlexia because of their superior word recognition ability. In addition, in alphabetic languages like English, the letter sound mapping of hyperlexic children seem to be intact (e.g., Healy, 1982; Kennedy, 2003), meaning that they do not have difficulty in processing smaller (than single word) orthographic unit, while in TYH, the knowledge of sub-lexical orthographic structure and knowledge seem to be challenging. This may imply that the weakness in the orthographic awareness of the smaller orthographic units (character structures and components) might be a language-specific feature for hyperlexics speaking Chinese.

In terms of how meta-linguistic awareness skills support hyperlexic reading, the study found that orthographic awareness skill is of similar importance in the reading of the hyperlexic child as in CA matched TD children. However, the hyperlexic child does not rely on morphological awareness skill in reading as much as CA matched children possibly due to the difficulty in language comprehension (Nation et al., 2006). The predictability of the phonological awareness skill was also different from TD children of similar mental ability, indicating in the reading of the hyperlexic child, meta-linguistic awareness support reading differently from TD children of similar mental ability. Taken together, the hyperlexic does not read in the same way as children of similar mental ability. His reading was similar to the CA group in the level of support from the orthographic awareness skills, while differ in the less reliance on the morphological awareness skills compared with the CA control. His orthographic awareness skill also differs from TD children which suggest larger support from the whole character recognition and less from sub-lexical orthographic knowledge.

The findings from the study add to the knowledge on hyperlexic reading from Chinese language perspective. Language universal and language-specific features of meta-linguistic awareness in hyperlexic reading were identified, and it is possible that the hyperlexic reading is achieved through the direct mapping between the whole character and the sound. However, the current study did not find direct evidence for reading through the direct mapping in hyperlexic reading, and this assumption yield further examination in the reading of hyperlexic children speaking Chinese as well as alphabetic languages.

There are several limitations of the study that should be taken into consideration when interpreting the results. Firstly, the study is a single-case study, which means that the findings may not be able to generalize to a larger population. More cases are needed to be reported in order to have a comprehensive understanding of how meta-linguistic awareness skills contribute to hyperlexic reading. Also, the study only included TD control groups matched for chronological age and mental ability. An additional TD control group matched in reading level would allow for further investigation on how the hyperlexic child perform in the meta-linguistic awareness skills in comparison with TD children of similar reading level. In addition, the TD groups were not matched with the hyperlexic child in gender, future study looking at hyperlexia can further examine the reading pattern and meta-linguistic awareness skills in comparison with better-controlled TD groups.

## 5. Conclusion

Findings from the current study suggest hyperlexic reading are supported by good lexical knowledge but does not necessarily require good awareness of sub-lexical orthographic structure of characters and advanced morphological awareness skill. Weakness in distinguishing meanings using context information might be related to the language comprehension difficulty. It is possible that reading aloud in Chinese-speaking hyperlexic is achieved through the direct mapping between whole character and its pronunciation (Glosser et al., 1996).

## Data availability statement

The original contributions presented in the study are included in the article/supplementary material, further inquiries can be directed to the corresponding author.

## Ethics statement

The studies involving human participants were reviewed and approved by The University of Hong Kong Human Research Ethics Committee. Written informed consent to participate in this study was provided by the participants' parents or caregiver.

## Author contributions

LL and I-FS contributed to the conception and design of the study and interpreted the data. LL organized the data collection and performed the statistical analysis under the supervision of I-FS. LL wrote the first draft of the manuscript. All authors contributed to the article and approved the submitted version.

## Conflict of interest

The authors declare that the research was conducted in the absence of any commercial or financial relationships that could be construed as a potential conflict of interest.

## Publisher’s note

All claims expressed in this article are solely those of the authors and do not necessarily represent those of their affiliated organizations, or those of the publisher, the editors and the reviewers. Any product that may be evaluated in this article, or claim that may be made by its manufacturer, is not guaranteed or endorsed by the publisher.
